# Water-Accelerated Decomposition of Olefin Metathesis Catalysts

**DOI:** 10.1021/acscatal.2c05573

**Published:** 2023-01-03

**Authors:** Christian O. Blanco, Deryn E. Fogg

**Affiliations:** † Center for Catalysis Research & Innovation and Department of Chemistry and Biomolecular Sciences, University of Ottawa, Ottawa, Ontario, Canada K1N 6N5; ‡ Department of Chemistry, University of Bergen, Allégaten 41, N-5007 Bergen, Norway

**Keywords:** olefin metathesis, bimolecular decomposition, β-hydride elimination, ruthenium, aqueous metathesis, chemical biology

## Abstract

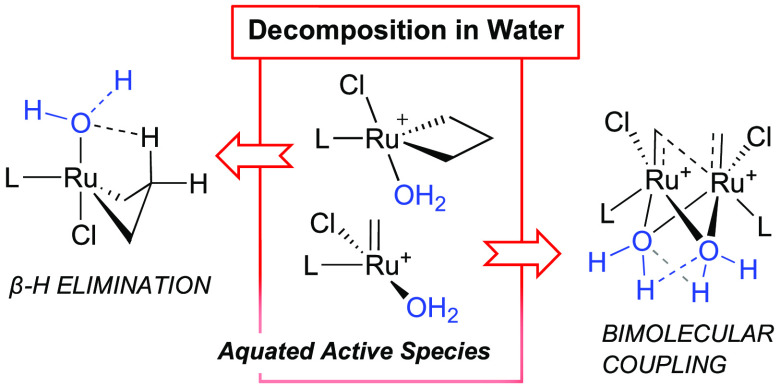

Water is ubiquitous in olefin metathesis, at levels ranging from contaminant to cosolvent. It is also non-benign. Water-promoted catalyst decomposition competes with metathesis, even for “robust” ruthenium catalysts. Metathesis is hence typically noncatalytic for demanding reactions in water-rich environments (e.g., chemical biology), a challenge as the Ru decomposition products promote unwanted reactions such as DNA degradation. To date, only the first step of the decomposition cascade is understood: catalyst aquation. Here we demonstrate that the aqua species dramatically accelerate both β-elimination of the metallacyclobutane intermediate and bimolecular decomposition of four-coordinate [RuCl(H_2_O)_n_(L)(=CHR)]Cl. Decomposition can be inhibited by blocking aquation and β-elimination.

Olefin metathesis is an exceptionally versatile catalytic means of forging carbon–carbon bonds,^1,2^ with frontier applications spanning pharmaceutical manufacturing,^3^ materials science,^4^ and chemical biology.^5,6^ Water is ubiquitous in all of these contexts, at levels ranging from contaminant to cosolvent. This is important because water is now known to limit metathesis productivity even for relatively robust ruthenium catalysts (Chart 1). For demanding reactions in water-rich media (e.g., protein modification via cross-metathesis (CM)^6^ or assembly of DNA-encoded libraries (DEL) via ring-closing metathesis^5c,5e^ (RCM)), the ruthenium complex must be used in significant stoichiometric excess, in part due to water-induced decomposition. Catalysis has been achieved only where the ruthenium complex is shielded in a lipophilic region.^5a,5b,5d^ Catalytic metathesis is desirable not merely for efficiency: in certain chemical biology applications (DEL being a prominent recent example), it is critical, because the spent catalyst triggers DNA degradation.^5c^ More broadly, understanding how water promotes decomposition of ruthenium metathesis catalysts is critical to expand opportunities in these and other forefront applications, in which water is either necessarily present or impractical to remove.

**Chart 1:**
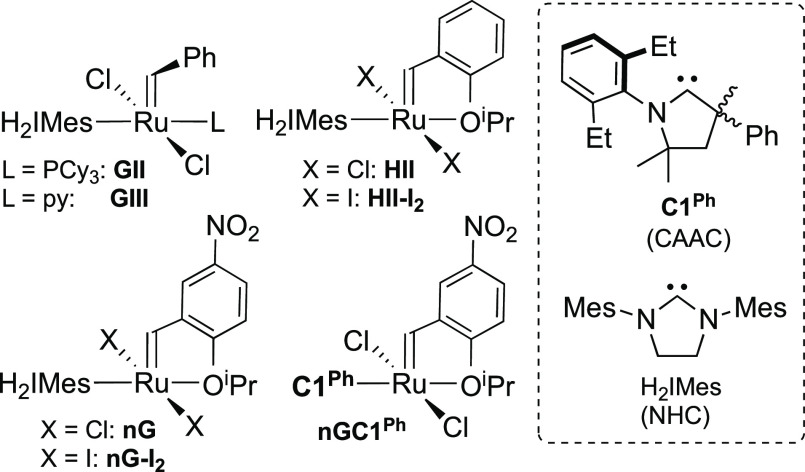
Olefin Metathesis Catalysts Discussed.a

a **GIII** is a mono-/bis-pyridine mixture; for convenience, only the former is shown.^7^

Metathesis in water has been described as a race against decomposition.^6c^ Productivity is low and declines as the proportion of water increases. For example, a record turnover number (TON) of 640 was reported for a ruthenium-cyclic (alkyl)(amino)carbene (CAAC) catalyst for metathesis in 1:1 H_2_O-MeOH: in neat water, TONs dropped to 200.^8^ In stark contrast, TONs up to 350 000 have been achieved in anhydrous solvent.^9^ For Ru-H_2_IMes catalysts, TONs for (R)CM in water are <100.^10,11^ The O’Reilly and Matsuo teams drew the logical inference that the ruthenium species present in aqueous solution are both less active and less stable.^12,13^ Decomposition of **Ru-1** (Scheme 1a) or **GIII** was shown to commence with formation of an aqua complex (observed by UV-vis analysis at high water concentrations), with the ensuing pathways remaining speculative. Scheme 1a depicts the initial equilibrium (a classic aquation-anation exchange, where anation signifies replacement of bound water by the chloride counteranion).^14^ Consistent with decomposition via an aqua species,^15^ metathesis activity was restored by addition of chloride salts routinely used to shift the aquation equilibrium in biological media.^16^

**Scheme 1:**
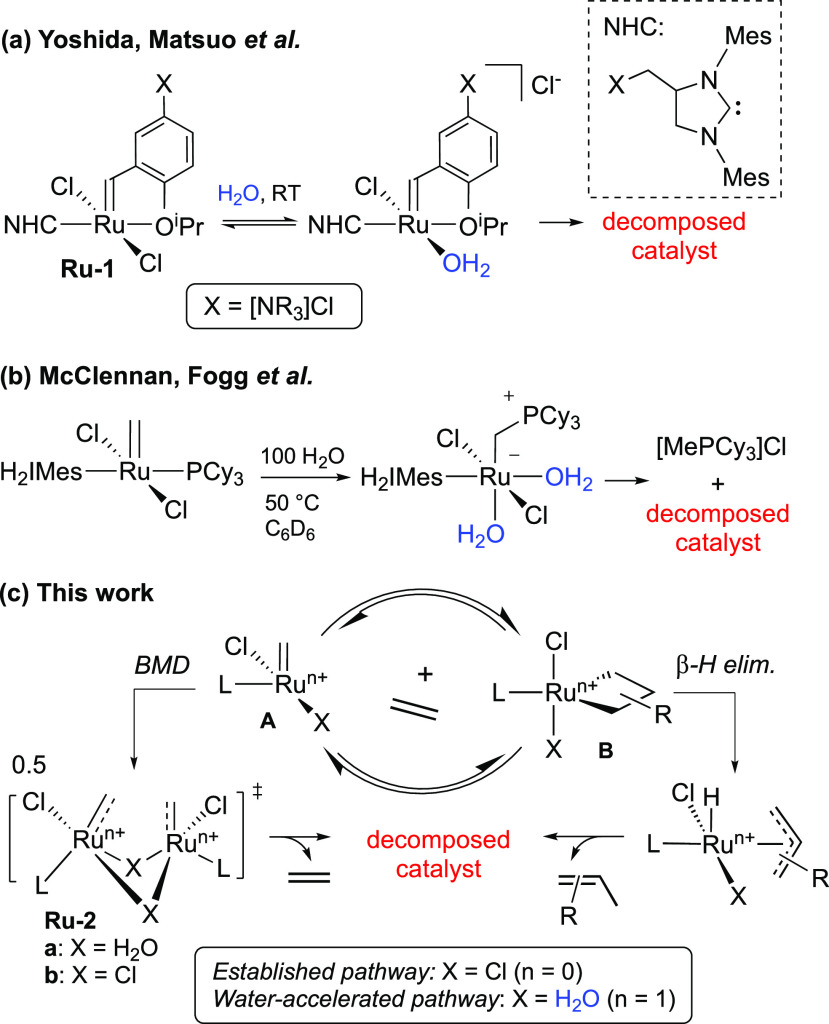
(a) Aquation-Initiated Catalyst Decomposition in Bulk Water. (b) Donor-Accelerated Decomposition of **GII**. (c) Water-Accelerated Degradation of Phosphine-Free Catalysts (Proposed)

The challenges associated with metathesis in the presence of *trace* water (important for both bench-scale^17^ and process^3^ chemistry) have been viewed as an independent problem. Metathesis productivity is severely degraded even at low concentrations of water, including the micromolar limits encountered in water-immiscible aromatic solvents (standard media for metathesis).^18−22^ It is unclear, however, whether aquation is relevant. The sole class of metathesis catalysts subjected to mechanistic study under such conditions are phosphine-stabilized Grubbs complexes, such as **GII**. Quantitative elimination of [MePCy_3_]Cl from **GII** in benzene-water supports an alternative mechanism, “donor-accelerated decomposition” (Scheme 1b).^23^ That is, coordination of water accelerates loss of PCy_3_, which then abstracts the methylidene ligand as [MePCy_3_]Cl.

Methylidene abstraction, however, is driven by the powerful nucleophilicity of PCy_3_.^24^ This mechanism is irrelevant to the decomposition of *phosphine-free* catalysts such as **HII**, but the latter are no less vulnerable than **GII** in “wet” aromatic solvents^19−21^ (indeed, fast-initiating variants are significantly more so).^19^ We therefore set out to clarify the pathways by which water degrades phosphine-free catalysts, with an explicit focus on the mechanism operative at low proportions of water. We considered two limiting possibilities: (1) that decomposition would proceed via aquation as in Scheme 1a, at a slower rate (in which case identification of post-aquation deactivation steps would be relevant to decomposition by both bulk and trace water) or (2) that aquation would be too slow to contribute, enabling an alternative pathway to take over. Here we report evidence for aquation even at millimolar concentrations of water and present the first insights into the ensuing decomposition pathways. Water is shown to accelerate two pathways operative in anhydrous media: bimolecular decomposition (BMD) of methylidene species **A** and β-H elimination of the metallacyclobutane intermediate **B** (Scheme 1c).

We began by examining whether decomposition follows a different mechanism in water-saturated benzene^18^ or if the pathway established in bulk water remains relevant. Building on the premise that decomposition commences with aquation and that added chloride salts inhibit this equilibrium reaction,^15^ we examined metathesis productivity in benzene-water and the extent to which it is mitigated by added chloride ion. If aquation does not contribute to decomposition, we reasoned that TONs should be unaffected. As substrate, we chose *N*-tosyl diallylamine **1**, the facile cyclization of which permits low catalyst loadings (50 ppm = 0.005 mol %; 5 μM Ru), hence providing an aggressive test of the impact of water.

The radar plots of Figure 1 give a straightforward visual comparison of the impact of water and chloride salt. The outermost (dashed) line indicates the maximum attainable TON of 20 000; deviations toward the center signify lower productivity. While RCM is near-quantitative for all catalysts but **GII** in dry benzene (gray line), TONs drop significantly in the presence of water (blue line). Maximum negative impact was seen for **nG** and **HII**,^25^ and least for the iodide and CAAC catalysts, consistent with our prior report.^19^ Addition of KCl had a beneficial impact in all cases (in the case of **HII**, TONs are tripled), but in no case is activity completely restored to anhydrous levels. This is unsurprising, given restrictions on partitioning of KCl into the organic phase. Clearly, however, the positive effect of chloride established in bulk water is maintained at the lower proportions of water examined herein. These data point toward aquation as an important initial step in water-induced degradation even when the catalyst is confined to the organic phase.

**Figure 1:**
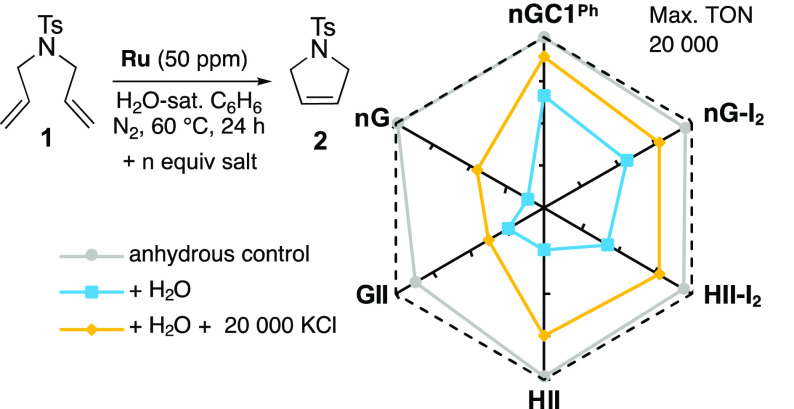
Effect of KCl on catalyst productivity^28^ in oxygen-free, water-saturated benzene with efficient volatilization of ethylene (see Supporting Information (SI)). Numerical values appear in Table S1; ±2 in replicate runs. Addition of N^*n*^Bu_4_Cl was less effective: see Figure S1.

The increase for **nGC1**^**Ph**^ holds more specific mechanistic significance. Whereas Ru-NHC catalysts undergo both decomposition pathways shown in Scheme 1c, an exceptional feature of the CAAC catalysts is their immunity to β-H elimination, but not bimolecular coupling. The higher productivity of **nGC1**^**Ph**^ in the presence of added KCl thus implies that water-promoted BMD proceeds via an aqua complex. Also noteworthy is the resistance of the iodide complexes to decomposition by water. This may reflect the greater covalency of the Ru–iodide bond, which has been found to inhibit aquation in other contexts.^26^ The improved performance declines over time, however, owing to competing halide exchange with KCl.^27^

We previously suggested^19^ that the deleterious effects of water in metathesis primarily involve the active species, as also established in decomposition by nucleophiles and Bronsted base.^29−31^ Countering this hypothesis is the reported decomposition of the precatalysts **Ru-1** and **GIII** in bulk water, as noted above. To resolve the discrepancy, we examined the stability of a series of precatalysts in anhydrous and “wet” benzene. In these experiments, the Ru complexes were stirred at 60 °C without substrate: decreases in the intensity of the benzylidene signal vs internal standard (IS) were monitored for 48 h or until decomposition was complete.

Py-stabilized **GIII** was completely decomposed after 4 h in water-saturated benzene, as compared to just 9% in the anhydrous control reaction (Table 1, entries 1, 2). Indeed, in the absence of water, 25% **GIII** remains even after 5 days at 60 °C.^7^ Water has less impact on **GII** (entry 3), consistent with the low lability of PCy_3_.^32^ For both **GIII** and **GII**, however, quantitative elimination of stilbene (Figure S6) offers unequivocal evidence that water degrades these complexes by accelerating bimolecular decomposition.^7,33^ (Nucleophilic attack of PCy_3_ on the substituted alkylidene carbon is not observed, in contrast with the facile abstraction of *methylidene* ligands shown in Scheme 1b.^23^) Equilibrium binding of water to form a spectroscopically unobservable,^34^ organic-soluble aqua complex is proposed to accelerate dimerization via intermolecular H-bonding (see **Ru-2a**, Scheme 1c).^35^ The chloride-bridged dimer **Ru-2b** was identified in computational studies of anhydrous BMD.^7^

**Table 1:**
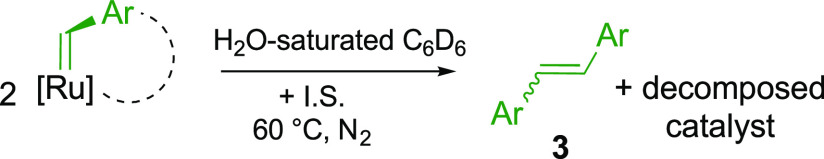
H_2_O-Induced Degradation of Precatalysts

Entry	Catalyst	*t* (h)	% [Ru]=C*H*Ar^a^^,^^b^	% Stilbene^b^
1	**GIII**	2	34 (96)	66 (4)
2	**GIII**	4	0 (91)	100 (9)
3	**GII**	4	54 (100)	46 (100)
4	**GII**	24	0 (100)	100 (0)
5	**nG**	48	>98 (>98)	0 (0)
6	**HII**	48	>98 (>98)	0 (0)
7	**HII–I**_**2**_	48	>98 (>98)	0 (0)
8	**nG-I**_**2**_	48	>98 (>98)	0 (0)
9	**nGC1**^**Ph**^	48	>98 (>98)	0 (0)

a [Ru] = RuX_2_(L); L = H_2_IMes or C1^Ph^; X = Cl or I.

b In brackets: anhydrous control. ±2% in replicate runs.

Neither **HII** nor **nG** underwent degradation after 2 days at 60 °C (entries 5, 6). In contrast, the water-soluble **HII** analogue **Ru-1** is reported to be completely decomposed after 16 h at RT in neat H_2_O (Scheme 1a).^12^ We attribute the difference to the high concentrations of Ru and water under the literature conditions and a resulting shift of the aquation-anation equilibrium to the right.

Bimolecular coupling is extremely sensitive to steric bulk.^7,33^ Given that water dramatically accelerates coupling of the benzylidene precatalysts **GII** and **GIII**, we anticipated that BMD would be the primary vector for catalyst decomposition in metathesis of terminal olefins in benzene-water, a reaction manifold propagated by methylidene species **A** (Scheme 1c, left). Unexpectedly, however, the rate of decomposition does not show a squared dependence on [Ru]; rather, it lies between first and second order (Figure S3). We thus questioned whether water also promotes a second major decomposition pathway intrinsic to the majority of olefin metathesis catalysts: β-H elimination of the metallacyclobutane **B** (Scheme 1c, right).^36^ To assess the impact of water on the latter pathway, we quantified the propenes eliminated during self-metathesis of styrene (Figure 2). Styrene is an invaluable substrate for this purpose because—unlike other 1-alkenes—it cannot isomerize to generate “false” propene markers.^7^ Propene yields were assessed by quantitative ^1^H NMR analysis at 24 h,^37^ to ensure full catalyst decomposition.

**Figure 2:**
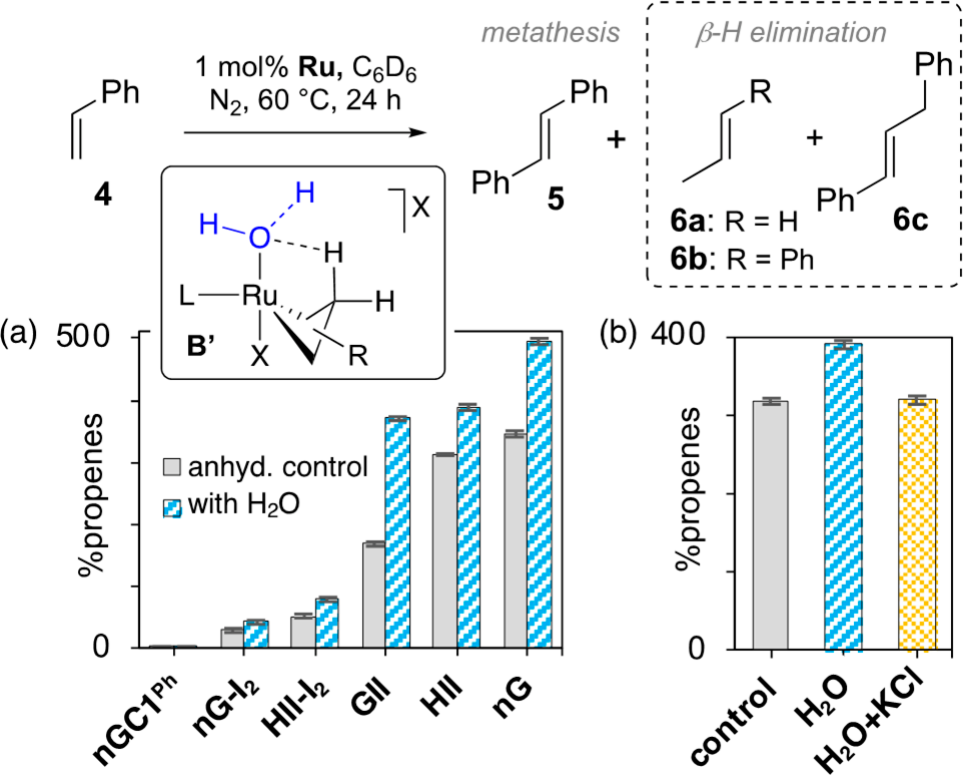
Impacts of H_2_O or KCl on β-H elimination from the putative aqua complex **B′** (X = Cl, I). (a) Acceleration by H_2_O. (b) Inhibition by KCl; shown for **HII**. For tabulated data, see SI. All reactions were carried out under oxygen-free conditions, with efficient removal of ethylene.

No propenes (**6a−c**, Figure 2a) were detected for the CAAC catalyst **nGC1**^**Ph**^ in these experiments. In earlier studies conducted under anhydrous conditions, we reported that the CAAC catalysts, exceptionally, resist β-H elimination,^38^ because the high trans-influence of the carbene destabilizes the transition state for decomposition.^39^ The absence of propenes for **nGC1**^**Ph**^ indicates that water is unable to overcome this resistance. In contrast, the yields of **6a**–**c** for the NHC catalysts increase in the presence of water, as indicated by the other data in Figure 2a. Hydrogen-bonding interactions between bound water and H_β_ (see **B′**) are proposed to aid in the H-transfer step. The effect is greatest for **HII** and **nG**, which spend the most time in the active cycle. The inhibiting effect of added chloride on production of propenes (shown for **HII** in Figure 2b) is consistent with the involvement of an aqua species in the transition state for β-H elimination.

Among the NHC complexes, the bis-iodide catalysts (**nG-I**_**2**_, **HII-I**_**2**_) underwent the least water-promoted β-H elimination, as anticipated from their relative water-tolerance.^19,40,41^ This heightened stability may reflect the resistance of the Ru–I bond to aquation, as discussed above.^42^ Also of note are changes in the distribution of propene products in the presence of water. Higher yields of substituted **6b/c** vs **6a** (approximately double; Figure S2) could plausibly be due to faster reductive elimination from **B′**, owing to increased steric pressure by the iodide ligands on the substituted metallacyclobutane.

An unexpected feature of these experiments is the superstoichiometric yield of propenes with respect to the initial catalyst charge. We infer regeneration of active catalyst from decomposed ruthenium species. In situ installation of an alkylidene ligand—standard practice in ROMP prior to development of “well-defined” metathesis catalysts^43,44^—has recently been demonstrated for simple 1-olefins.^45^ This observation holds considerable interest for the goal of recycling spent catalyst into the active cycle.^45,46^ Nevertheless, the key point in the present context is the increase in propene yields in the presence of water, signifying enhanced β-H elimination.

Water has long presented an under-recognized challenge to metathesis reactions. Expanding interest in applications where water is inevitable has turned a spotlight on ruthenium–water interactions and their role in catalyst decomposition. In bulk water, chloride ion improves the productivity of Ru metathesis catalysts by reversing aquation. The foregoing establishes that aquation occurs even at low water concentrations (i.e., the same mechanism is operative in bulk and trace water) and offers the first insights into the ensuing decomposition cascade. The water ligand greatly accelerates two decomposition pathways operative even in anhydrous media: bimolecular decomposition of the methylidene intermediate and β-H elimination of the metallacyclobutane.^47^ Recent discoveries that teach how to curb these pathways hence open the door to *catalytic* metathesis for demanding reactions in water. A high trans-influence ligand remains the key to inhibiting β-elimination; classic anchoring strategies, or aquation-resistant ligands, will aid further. These insights are anticipated to create major new opportunities in chemical biology and other contexts where water is essential.

## Supporting Information Available

Experimental and computational details; NMR spectra (PDF)
