# Supplementary material for: Water-Accelerated Decomposition of Olefin Metathesis Catalysts
Source: ACS Catal. 2023 Jan 3;13(2):1097–102. doi: 10.1021/acscatal.2c05573 (PMC9872090; doi:10.1021/acscatal.2c05573)
Supplement: Supplementary file 1 — cs2c05573_si_001.pdf [file cs2c05573_si_001.pdf]

Supporting Information

Water-Accelerated Decomposition of Olefin Metathesis Catalysts

Christian O. Blanco[a] and Deryn E. Fogg*[a,b]

[a]Center for Catalysis Research & Innovation, and Department of Chemistry and Biomolecular Sciences, University of Ottawa, ON, Canada, K1N 6N5. [b]Department of Chemistry, University of Bergen, Allégaten 41, N-5007 Bergen, Norway

*Corresponding author: dfogg@uottawa.ca, dfo025@uib.no

Table of Contents

S1. Experimental S2
S1.1. General Procedures. S2
S1.2. Synthesis of *N*-(*p*-toluenesulfonyl-2,5-dihydropyrrole), **2**. S2
S1.3. Examining Impact of H_2O and Chloride Salts on Metathesis S3
S1.4. Examining Stability of Precatalysts to H_2O S4
S1.5. Assessing Decomposition via H_2O-Accelerated β-H Elimination S5
S1.6. UV-Vis Analysis of the Decomposition Kinetics of **HII** in the Presence of H_2O S8
S2. NMR Spectra and GC Trace. S9
S3. References. S17

S1. Experimental

S1.1. General Procedures. All reactions were carried out in an N_2-filled glovebox unless otherwise noted. HPLC-grade THF and C_6H_6 was dried and degassed with a Glass Contour solvent purification system and stored under N_2 over 4 Å molecular sieves for at least 24 h prior to use. A water content of ≤4 ppm was confirmed by Karl-Fischer titration prior to use. Metathesis catalysts (**GII**,[1] **GIII**,[2] **nG**,[3] **HII**,[4] and **HII-I₂**,[5] Chart S1) and *N*,*N*-diallyl-4-methylbenzenesulfonamide **1**[6] were prepared by literature methods. Styrene (**4**; Aldrich, 99%), dodecane (Sigma, anhydrous, 99%; GC internal standard), C_6D_6 and D_2O (Cambridge Isotopes) and H_2O (deionized but not otherwise purified) were freeze-pump-thaw degassed (4×) and stored under N_2 in the glovebox. C_6D_6 was stored over sieves as above; styrene and **1** were kept at –35 °C. Dimethyl terephthalate (DMT) and anthracene (internal NMR standards; Sigma, 99%), potassium trispyrazolyl borate (KTp; quenching agent;[7] Sigma, 98%), **nG-I₂** and **nGC1^{Ph}** (kindly provided as gifts by Apeiron Synthesis) were used as received. The purity of all catalysts was confirmed by ^{1}H NMR analysis prior to use. For accuracy, solid catalysts were weighed outside the glovebox using a microanalytical balance. Metathesis reactions were carried out in sealed vials with good stirring and sufficient headspace (e.g. 2 mL solvent in a 4 mL vial) to promote efficient volatilization of ethylene. Control experiments carried out using vials open to the glovebox atmosphere show yields within 0-1% difference.

NMR spectra were recorded on Bruker Avance and Avance II 300 MHz NMR spectrometers at 25.0 ±0.5 °C. Chemical shifts (ppm) are referenced to the residual proton of the deuterated solvent. RCM reactions were analyzed using an Agilent 7890A gas chromatograph (GC) equipped with auto-sampler, flame ionization detector (FID) and Agilent HP-5 polysiloxane column (30 m length, 320 µm diameter). Column pressure was maintained at 11.5 psi with helium (UHP grade) as the carrier gas. Calibration curves of peak areas vs concentration were established for **1** and its cyclized product **2** in the relevant concentration regimes (1:1 w/w vs dodecane internal standard).

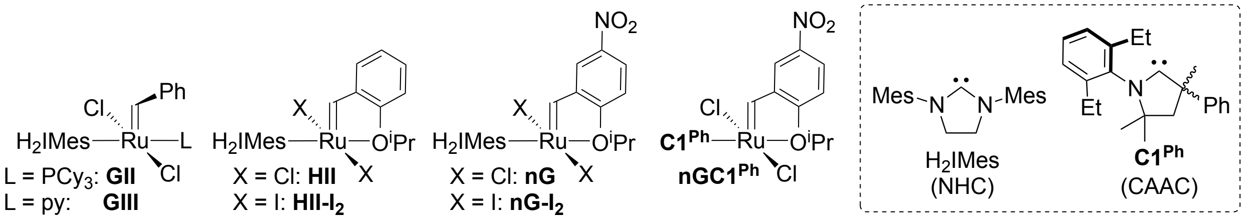

Chart 1. "Well-Defined" Catalysts Employed for Olefin Metathesis.

S1.2. Synthesis of *N*-(*p*-toluenesulfonyl-2,5-dihydropyrrole), 2. Compound **2** was required to establish a calibration curve for GC quantitation. As the *Org. Synth.* route,[8] though high-yielding (90-94%), requires 2.5 h reflux, a more convenient method was employed using 1 mol% **DA** instead of 0.5 mol% **GI**. RCM was then complete within minutes at RT in the glovebox; it was purified by chromatography rather than extraction and Kugelrohr distillation. Accordingly, solid green **DA** (9.0 mg, 1.2 mmol, 0.01 equiv, 1 mol%) was added to a solution of *N*,*N*-diallyl-4-methylbenzenesulfonamide **1** (0.300 g, 1.19 mmol, 1.00 equiv) in 12 mL CH_2Cl_2. The resulting yellow solution was stirred at RT for 20 min, at which point

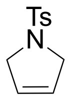

2

formation of **2** as the sole product was complete (GC-FID). The reaction was quenched with KTp (10 mg in 1 mL THF; 10 equiv vs **DA**),[7] concentrated to a green oil (rotovap), and purified by flash chromatography (10% EtOAc in hexanes; 10 × 1.5 cm column), to afford **2** as a white solid. Yield: 240 mg (1.07 mmol, 90%). The ^{1}H NMR data agree with literature values;[6] they are provided here for convenience.

^{1}H NMR (300 MHz, $CDCl_3$): δ 7.72 (d, $^3J_{HH}$ = 8.1 Hz, 2H, tosyl *o*-C*H*), 7.32 (d, $^3J_{HH}$ = 8.1 Hz, 2H, tosyl *m*-C*H*), 5.65 (s, 2H, =C*H*), 4.12 (s, 4H, =CHCH_2N), 2.43 (s, 3H, tosyl CH_3). Purity was confirmed by GC-FID (>99.8%). See Figure S4 in Section S2.

S1.3. Examining Impact of H_2O and Chloride Salts on Metathesis

S1.3.1. Representative Procedures: RCM of *N*-Tosyl Diallylamine (1) in the Presence or Absence of H_2O

Anhydrous: Diene **1** (56 µL, 0.20 mmol) and dodecane (45 µL, 0.20 mmol, 1 equiv; internal standard for GC analysis) were dissolved in 1.82 mL C_6H_6; final concentration 200 mM **1**. A 50 µL aliquot was removed for GC-FID analysis to establish the starting ratio of **1** : dodecane. To the stirred solution at 60 ±1 °C (glovebox, degassed oil bath) was added catalyst, as an 78 µL aliquot of a stock solution of **HII** (10.4 mg **HII** in 1.0 mL C_6H_6, diluted 100-fold) to give a catalyst loading of 0.005 mol%. Aliquots were removed periodically, quenched with KTp in THF (10 mg/mL; 10 equiv vs starting Ru) and analyzed by GC-FID.

With water: As above, with addition of H_2O (20 µL, 1.1 mmol, 1% v/v) prior to catalyst.

With water and KCl: As above, adding KCl (14 mg, 0.20 mmol, 20 000 equiv) and H_2O (20 µL, 1.1 mmol, 1% v/v) prior to catalyst.

With water and N^nBu_4Cl: As above, adding N^nBu_4Cl (55 mg, 0.20 mmol, 20 000 equiv) and H_2O (20 µL, 1.1 mmol, 1% v/v) prior to catalyst.

Table S1 summarizes conversions, yields and TONs for all catalysts. Figure S1 depicts the impact of added chloride salts.

Table S1. Yields, TONs, and Impact of Water on RCM of **1** Using the Catalysts of Chart S1[a]

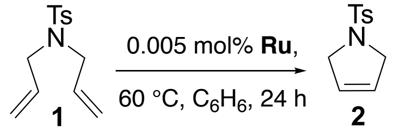

**Cat.**	**Conversion (%)**			**Yield (%)**			**TON**		
	**Dry**	**H_2O**	**KCl+H_2O**	**Dry**	**H_2O**	**KCl+H_2O**	**Dry**	**H_2O**	**KCl+H_2O**
**nG**	99	11	45	99	11	45	19 800	2 200	9 000
**HII**	99	24	76	99	21	75	19 800	4 200	15 000
**GII**	87	24	38	87	24	38	17 400	4 800	7 600
**HII-I$_2$**	94	43	77	94	43	76	18 800	8 600	15 200
**nG-I$_2$**	96	56	78	95	56	78	19 000	11 200	15 600
**nGC1^{Ph}**	100	66	91	100	66	89	20 000	13 200	17 800

[a]Numerical data for Fig. 2 in main text. Agreement in replicate run averages ±2%. Dry = anhydrous control reaction.

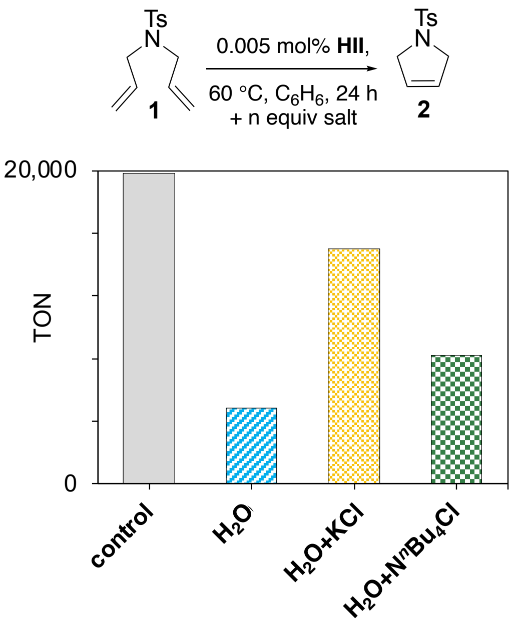

Figure S1. Impact of added chloride salts on RCM productivity.

S1.3.2. Confirming Halide Exchange of nG-I_2 with KCl on the Timescale of Metathesis.

Dark brown **nG-I_2** (10 mg, 0.012 mmol) and anthracene (ca. 1.0 mg) were dissolved in 1.5 mL C_6D_6 and stirred for 30 sec, after which a 0.5 mL aliquot was removed to establish the initial ratio of **nG-I_2** to anthracene (^{1}H NMR). To the dark-yellow solution was added H_2O (20 µL, 1.1 mmol, 1% v/v) and KCl (884 mg, 11.7 mmol, 1 000 equiv; incompletely dissolved). The heterogeneous mixture was stirred (glovebox, degassed oil bath) at 60 ±1 °C for 2 h, at which point an aliquot was removed for analysis. Partial conversion to **nG** (5%) and **nG-I** (29%) was observed. For starting and ending spectra, see Figure S5, Section 2.

S1.4. Examining Stability of Precatalysts to H_2O

S.1.4.1. Representative Procedure for Thermolysis of HII

Anhydrous. A solution of green **HII** (10 mg, 0.016 mmol) and DMT (ca. 1.0 mg) in 1.5 mL C_6D_6 was stirred for 30 sec, after which a 0.5 mL aliquot was removed to establish the initial ratio of **HII** to DMT (^{1}H NMR). Stirring was continued (glovebox, degassed oil bath) at 60 ±1 °C for 2 days, at which point an aliquot was removed for analysis.

With water: As above, adding H_2O (50 µL, 2.7 mmol, 174 equiv, 5% v/v) after the "t_0" aliquot.

The proportions of remaining catalyst ([Ru]=C*H*Ar) and the decomposition product stilbene **5** are given in Table 1 in the main text. Only **GII** and **GIII** formed stilbene **5** after thermolysis with and without water. In contrast, the Hoveyda-type catalysts **nG** and **HII** showed no decomposition even after heating in the presence of water for 48 h. Representative starting and ending spectra are given in Figure S6 (**GIII)** and S7 (**HII**).

S.1.4.2. Probing the Reactivity of PCy_3 Toward H_2O. These experiments were carried out in benzene-water, THF-water, or pure water. No reaction was observed.

In H_2O: Solid PCy_3 (10 mg, 0.035 mmol) in 1 mL H_2O was stirred (glovebox, degassed oil bath) at 60 ±1 °C. After 24 h, the solution was transferred to a septum-capped NMR tube and removed from the glovebox for NMR analysis. No PCy_3 protonation was evident by $^{31}P\{^1H\}$ NMR analysis (Figure S8a).

In THF: To a solution of PCy_3 (10 mg, 0.035 mmol) in 1 mL THF was added H_2O (50 µL, 2.77 mmol, 80 equiv) and the resulting homogeneous solution was stirred (glovebox, degassed oil bath) at 60 ±1 °C. After 24 h, the solution was transferred to a septum-capped NMR tube and removed from the glovebox for NMR analysis. No PCy_3 protonation was evident (Figure S9a).

In C_6H_6: As above. The solution was biphasic, owing to the limiting solubility of water in benzene (2 000 ppm at 60 °C). No PCy_3 protonation was evident (Figure S10a).

S.1.4.3. Protonation of PCy_3 by HCl. To the solutions above, HCl (30 µL of a 12 M solution, 0.036 mmol, 10 equiv) was added by injection through the septum, the NMR tube was shaken, and a $^{31}P\{^1H\}$ NMR spectrum was taken immediately. Complete conversion to $[HPCy_3]Cl$ was evident in water, THF, and benzene (Figures S8b, S9b, and S10b respectively).

S1.5. Assessing Decomposition via H_2O-Accelerated β-H Elimination

Decomposition of the metallacyclobutane intermediate was assessed from the yield of propenes generated during the self-metathesis of styrene **4**. These yields were measured by quantitative ^1H NMR (qNMR) analysis as described below.

S1.5.1. Measuring T_1 Relaxation Times for Propenes and HII. As the standard 0.01-sec longitudinal relaxation delay (d1) is insufficient for complete relaxation of the small molecule propene, the appropriate relaxation delay (≥5x T_1 for the slowest-relaxing signal of interest) was assessed by measuring T_1 values by the inversion-recovery method in C_6D_6. Table S2 summarizes T_1 and d1 values for the olefinic signals of **HII** and propenes **6a** and **6b**. As expected, the d1 value for **HII** is shortest (7.9 sec). More striking is the 5-fold longer D1 measured for propene **6a** vs its 1-phenylpropene congener **6b** (a manifestation of the exponential dependence of relaxation time on the correlation time). To ensure complete relaxation, qNMR was carried out with a 140-sec d1.

Table S2. T1 and d1 Values for **HII** and Propenes **6a** and **6b**

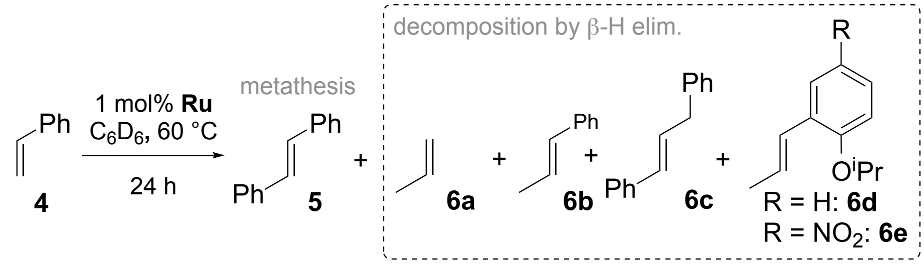

Compound	Irradiated Proton	Chemical shift	T_1 (s)	d1 (s)
**HII**	[Ru]=C*H*R	16.65	1.6	7.9
$CH_3HC=CH_2$, **6a**	=CH*H*	5.01	23.1	115.5
$CH_3HC=CHPh$, **6b**	=C*H*CH_2Ph	6.03	4.5	22.5

S1.5.2. Representative Procedure for Propene Quantitation

Anhydrous: Solid green **HII** (20 mg, 0.032 mmol) and DMT (1 mg, 0.051 mmol) were dissolved in 1.00 mL C_6D_6 to give a 32 mM stock solution of **HII**. A 384 µL aliquot (12 µmol **HII**) was transferred to a J-Young NMR tube and diluted with 1.86 mL C_6D_6. The initial ratio of **HII**:IS was measured, after which the NMR sample was returned to the glovebox and styrene **4** (137 µL, 1.2 mmol, 100 equiv) was added to give a solution 5 mM in **HII**. The NMR tube was transferred to an oil bath behind a blast shield and shaken vigorously every hour. The mixture was analyzed (NMR) after 24 h.

With water: As above, with addition of H_2O (24 µL, 1.3 mmol, 1% v/v) prior to catalyst.

For representative initial and final spectra, see Figure S11; for tabulated propene yields, see Table S3. The proportion of propene **6a** relative to the sum of all propenes is shown in Figure S2.

S1.5.3. Key ^{1}H NMR Data (C_6D_6, 300 MHz) for Propene Products[9]

H_2C=$CHCH_3$, 6a: δ 5.72 (m, 1H, =CH_XCH$_3$), 5.01 (m, 1H, =CH_AH$_B$; overlaps with =CH$_A$$H_B$), 4.95 (m, 1H, =CH$_A$$H_B$ overlaps with =CH_AH$_B$), 1.55 (dt, $^3J_{HH}$ = 6.4 Hz, $^3J_{HH}$ = 1.5 Hz, 3H, =CHCH_3).

H_2C=CHPh, 6b: δ 6.29 (dq, $^3J_{HH}$ = 15.8 Hz, $^4J_{HH}$ = 2.0 Hz, 1H, PhHC=), 6.03 (dq, $^3J_{HH}$ = 15.8 Hz, $^3J_{HH}$ = 6.7 Hz, 1H, =CHCH$_2$Ph), 1.65 (dd, $^3J_{HH}$ = 7 Hz, $^4J_{HH}$ = 2.0 Hz, 3H, =CHCH_3).

PhCH$_2$HC=CHPh, 6c: δ 6.31 (d, $^3J_{HH}$ = 16.0 Hz, 1H, PhHC=), 6.19 (dt, $^3J_{HH}$ = 16.0 Hz, $^3J_{HH}$ = 6.8 Hz, 1H, =CHCH$_2$Ph), 3.25 (d, $^3J_{HH}$ = 6.9 Hz, 2H, =CHCH_2Ph).

CH$_3$HC=CH(C$_6$H$_4$-2-O^{i}Pr), 6d: δ 6.66 (m, 1H, PhHC=), 6.16 (m, 1H, CH=CHPh), 4.18 (sept, $^3J_{HH}$ = 5.9 Hz, 1H, CHMe$_2$), 1.73 (dd, $^3J_{HH}$ = 6.6 Hz, $^4J_{HH}$ = 1.7 Hz, 3H, =CHCH_3), 1.09 (d, $^3J_{HH}$ = 5.9 Hz, 6H, ^{i}Pr CH_3).

S1.5.4. Yields of Propenes

Table S3. Propene Yields in Self-Metathesis of Styrene with Various Catalysts.[a]

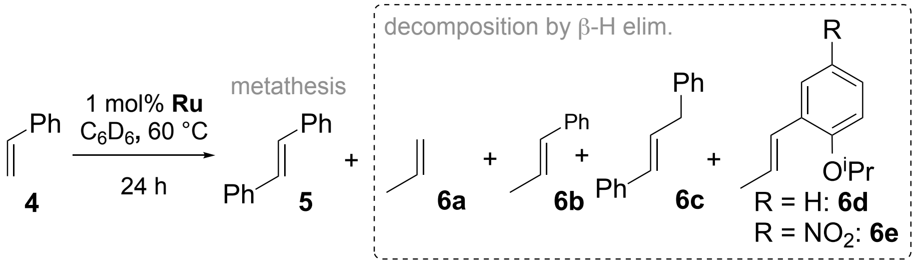

Cat.	Additive	% Yield in Reaction with H_2O (Anhydrous Yield)					$\Sigma_{anhyd} - \Sigma_{H_2O}$
		**6a**	**6b**	**6c**	**6d** or **6e**	Σ	
**nGC1^{Ph}**	-	<1 (0)	0 (<1)	0 (0)	0 (0)	<1 (<1)	0
**nG-I$_2$**	-	21 (41)	20 (13)	17 (0)	24 (0)	82 (53)	29
**HII-I$_2$**	-	16 (20)	20 (8)	16 (0)	18 (0)	66 (28)	28
**GII**	-	151 (94)	155 (97)	0	NA[b]	306 (191)	115
**HII**	-	260 (206)	133 (112)	0	0	393 (318)	81
**nG**	-	321 (232)	178 (114)	0	0	500 (346)	154
**HII**	KCl	207 (206)	112 (112)	0	0	319 (318)	1
**HII**	N^nBu_4Cl[c]	188 (206)	132 (112)	0	0	325 (318)	7

[a]Numerical data for Fig. 2 in main text. For error bars, see Fig. 2. [b]**GII** lacks a chelating benzylidene ligand and thus cannot form **6d**/**e**. [c]20 000 equiv.

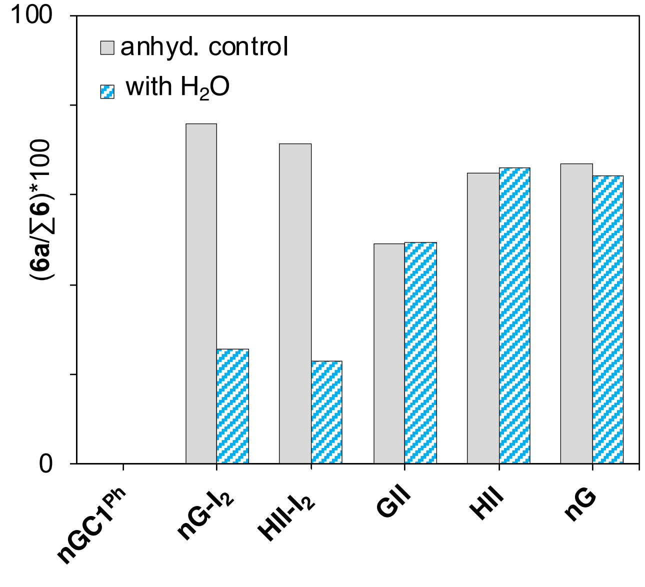

Figure S2. Proportions of propene formed by β-H elimination from the unsubstituted MCB, relative to sum of all propenes for various catalysts.

S1.6. UV-Vis Analysis of the Decomposition Kinetics of HII in the Presence of H_2O. Solid green **HII** (10 mg, 0.016 mmol) was dissolved in 1.00 mL C_6H_6 to give a 16 mM stock solution of **HII**. A 16 µL aliquot (256 nmol **HII**) was transferred to a quartz cuvette and diluted with 1.97 mL C_6H_6 (final concentration of **HII** 128 µM). To the solution was added H_2O (90 µL, 5 mmol, 1 000 equiv); the cuvette was capped and shaken vigorously. The initial absorbance of **HII** was measured and then styrene (133 µL from a stock solution of 20 mg/mL, 25.6 µmol,100 equiv) was added through the septum using a gas-tight syringe. The cell was shaken and returned to the spectrometer where spectra were taken every 5 minutes for 6 h (Figure S3a). All spectra were collected at 25 °C ± 0.01 °C. The decrease in absorbance as a function of time (Figure S3b) corresponds to decomposition of **HII**. The R^2 values in Figures S3c and S3d imply that both first- and second-order decomposition pathways are operative.

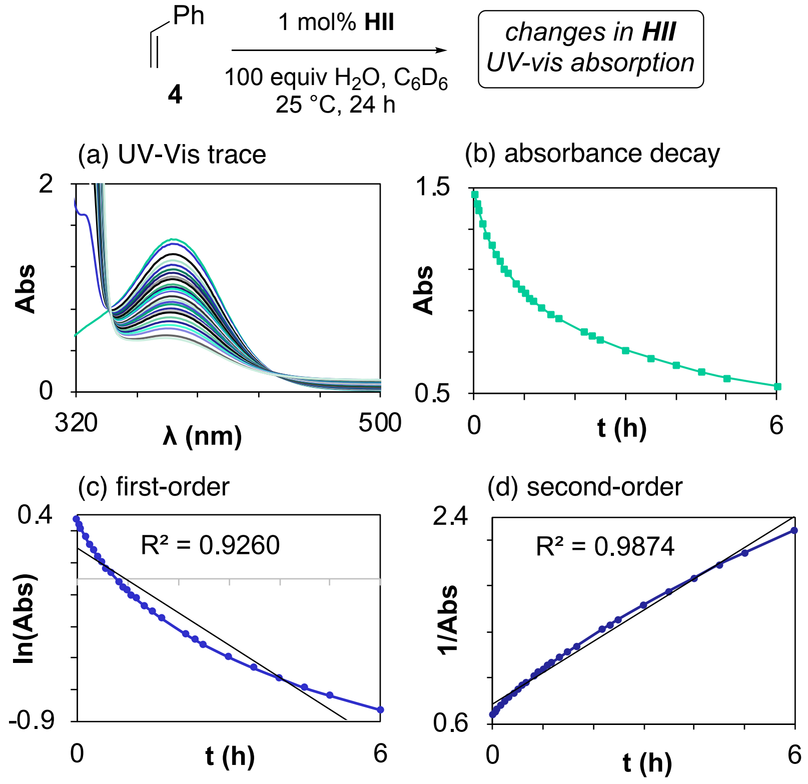

Figure S3. Decomposition of **HII** by water during the self-metathesis of styrene **4** at 25.0 °C. (a) UV-vis spectra measured every 5 min. (b) Decay in absorbance as a function of time. Kinetic plots: (c) first order, (d) second order.

S2. NMR Spectra and GC Trace.

(a) ^{1}H NMR

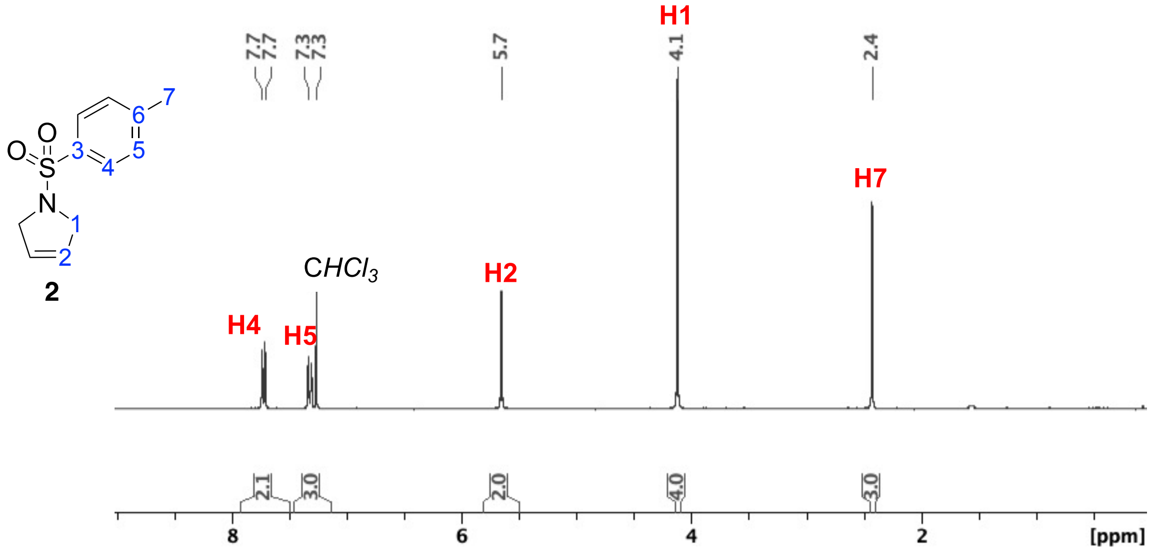

(b) GC-FID trace

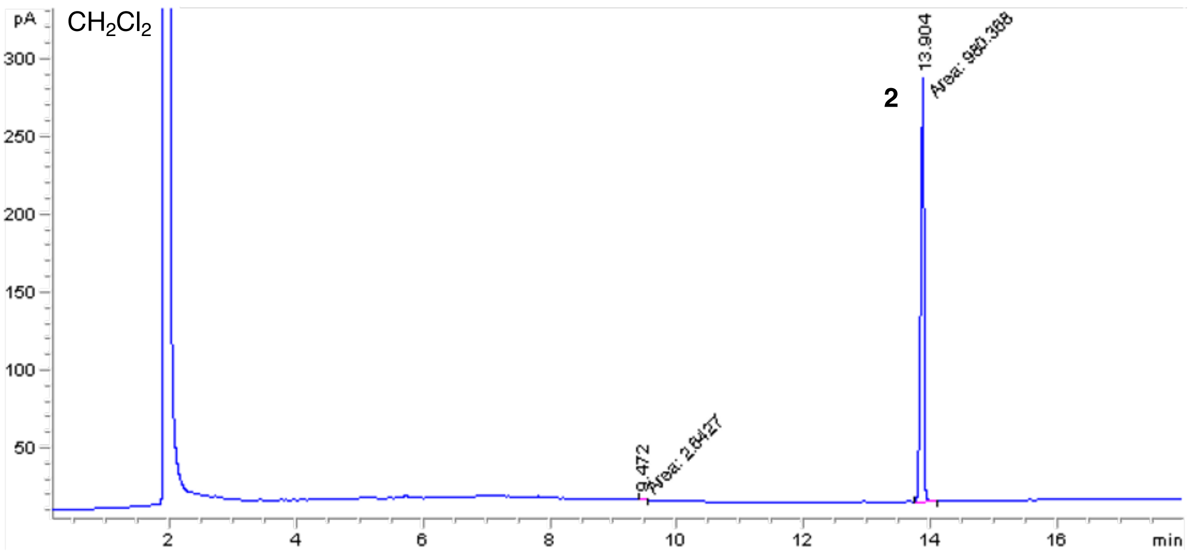

Figure S4. Characterization of cyclic *N*-(*p*-toluenesulfonyl-2,5-dihydropyrrole), **2**. (a) ^{1}H NMR spectrum (500 MHz, $CDCl_3$). (b) GC-FID trace showing >99% purity.

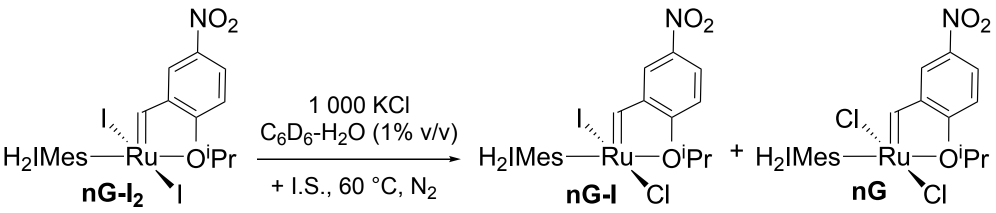

(a) t = 0 h

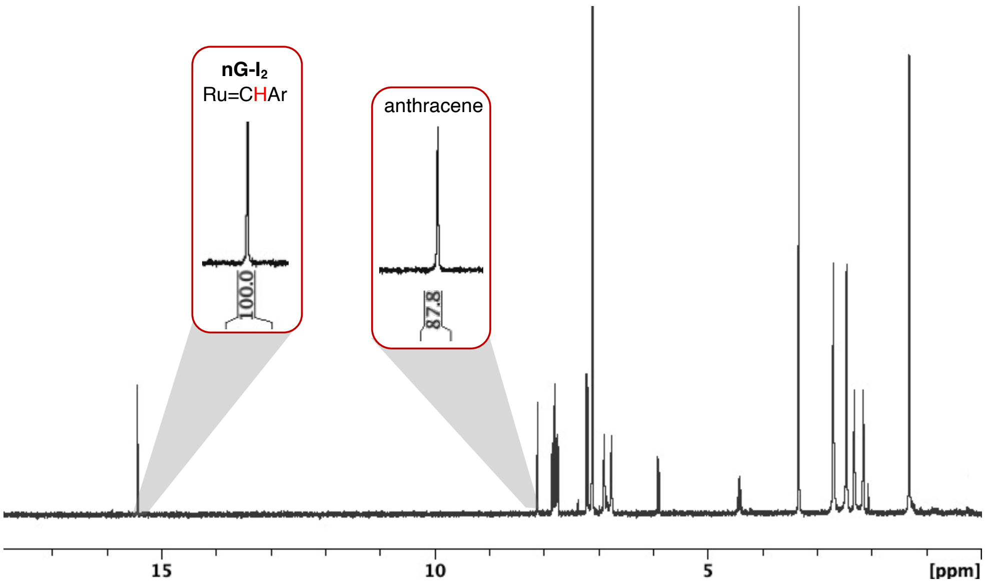

(b) t = 2 h

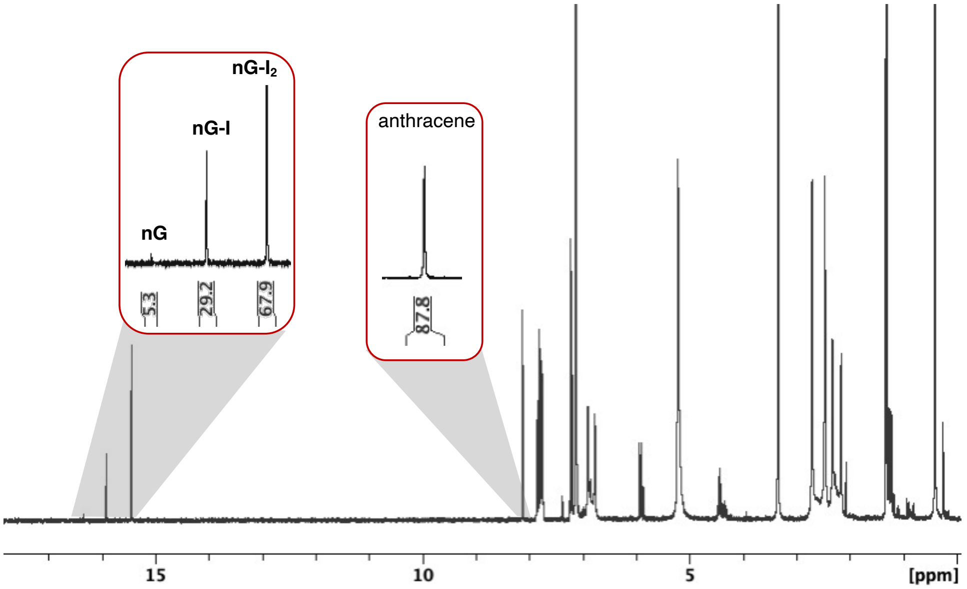

Figure S5. ^1H NMR spectrum (300 MHz, C_6D_6) showing partial conversion of **nG-I₂** by KCl water-saturated benzene. (a) At 0 h, before adding KCl. (b) After stirring with 1 000 equiv KCl at 60 °C for 2 h. Integrations of key signals (insets) are normalized to that of starting **nG-I₂.**

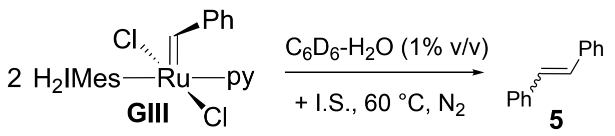

(a) t = 0 h

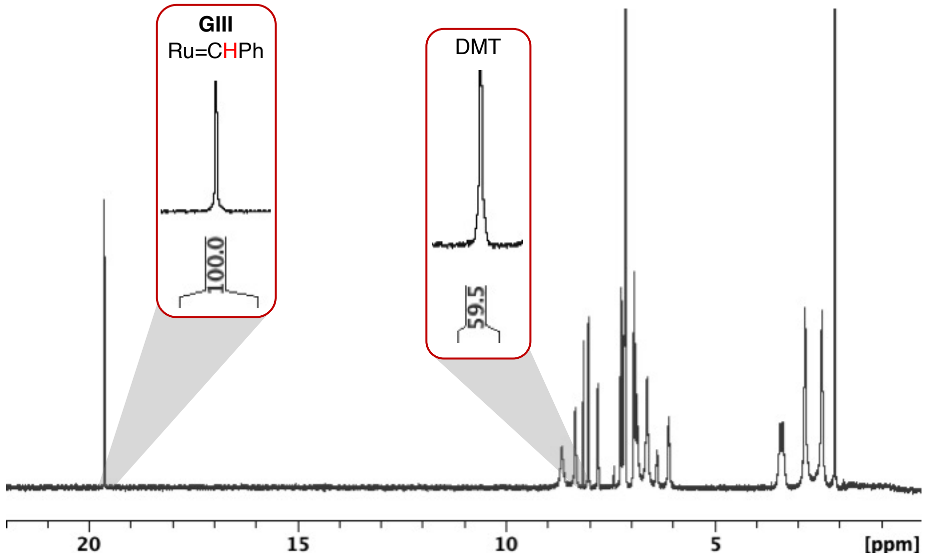

(b) t = 4 h

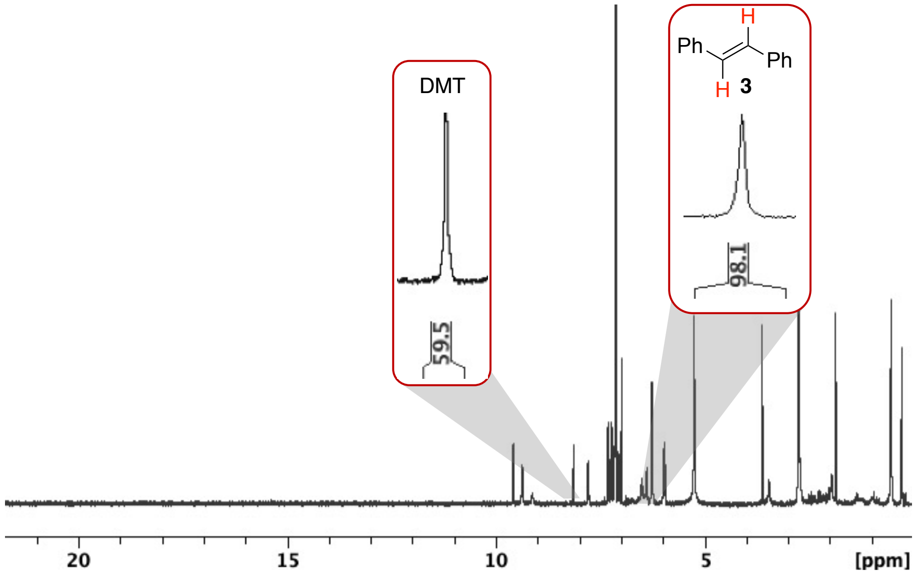

Figure S6. ^{1}H NMR spectrum (500 MHz, C_6D_6) showing full decomposition of **GIII** by water, and formation of stilbene **3**. (a) At 0 h, before heating to 60 °C. (b) After stirring for 4 h at 60 °C with 2% H_2O (v/v). Integrations of key signals (insets) are normalized to that of starting **GIII**.

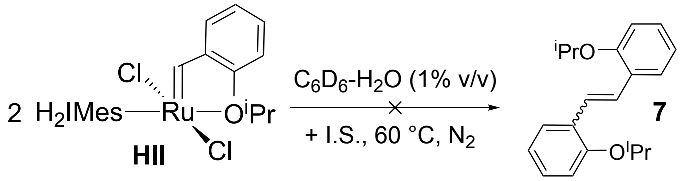

(a) t = 0 h

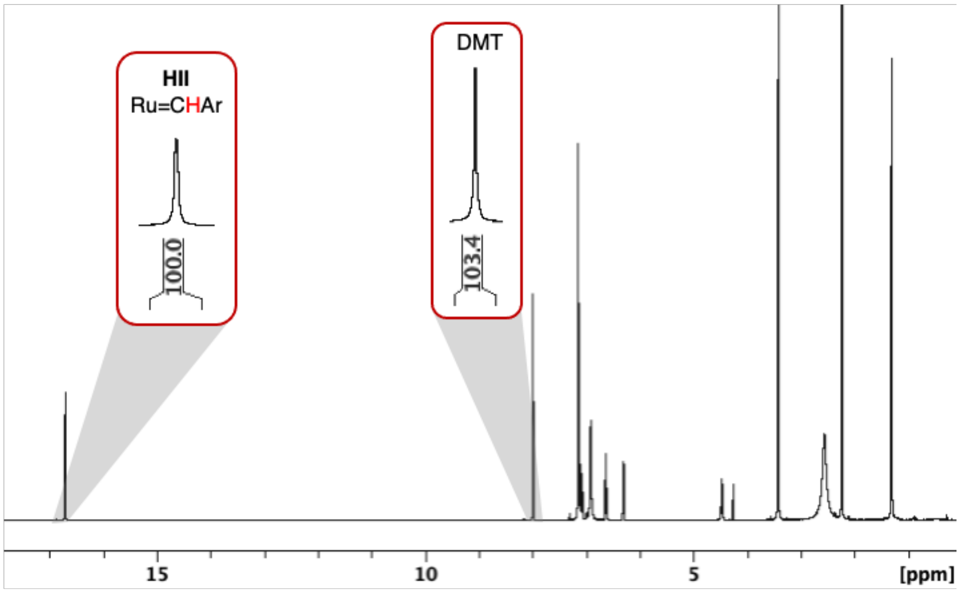

(b) t = 48 h

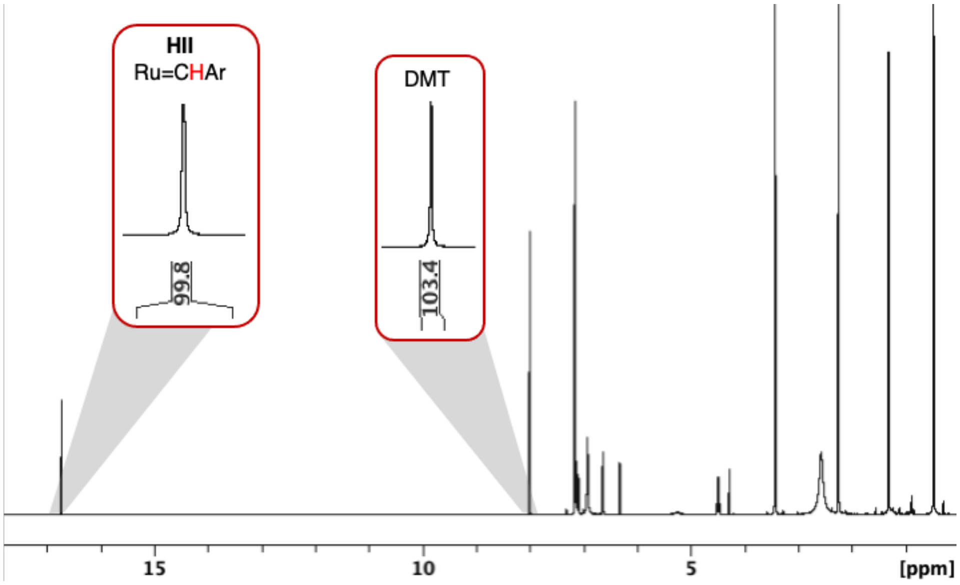

Figure S7. Stability of **HII** to thermolysis in the presence of water. ^{1}H NMR spectrum (500 MHz, C_6D_6). (a) At 0 h, before heating to 60 °C. (b) After stirring for 48 h at 60 °C with 2% H_2O (v/v). Integrations of key signals (shown in insets) are normalized to that of starting **HII**.

(a) Stability of PCy_3 to H_2O

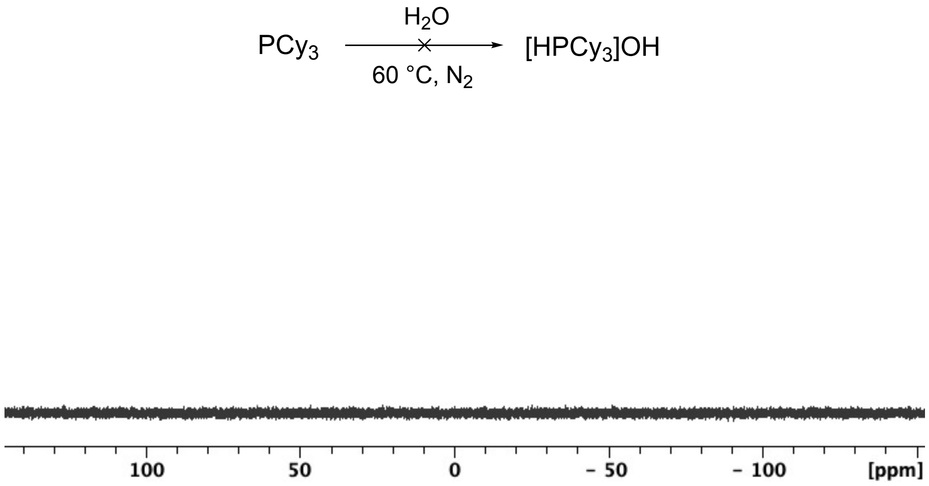

(b) Immediate reaction of PCy_3 with HCl in water

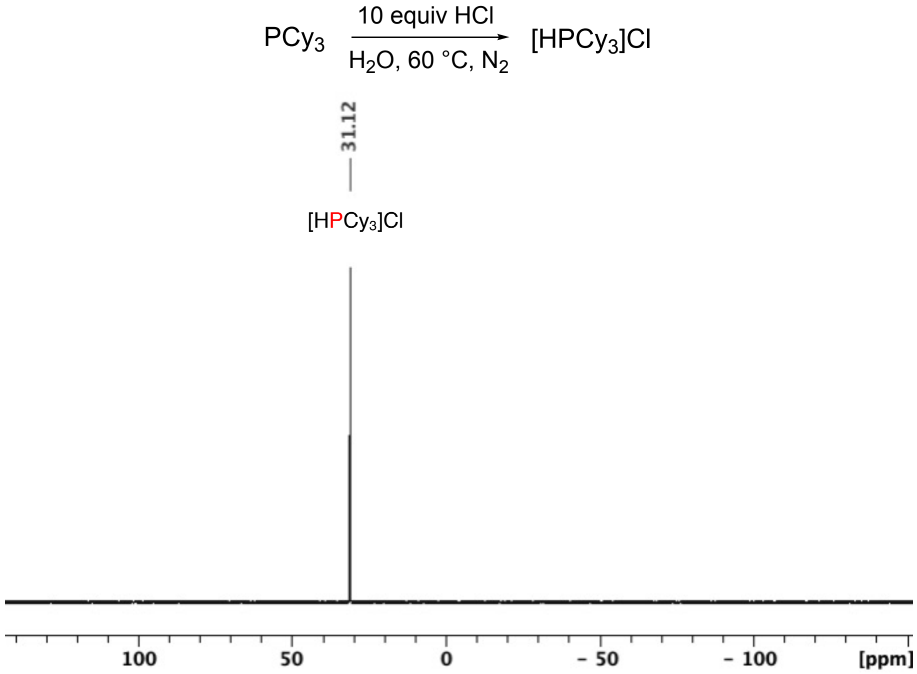

Figure S8. Assessing the reactivity of PCy_3 in water as solvent by $^{31}P\{^1H\}$ NMR analysis (121 MHz, H_2O). (a) Spectrum after heating in H_2O at 60 °C for 24 h. (b) Immediately after adding concentrated (12 M) HCl.

(a) Stability of PCy_3 to 5% H_2O in THF

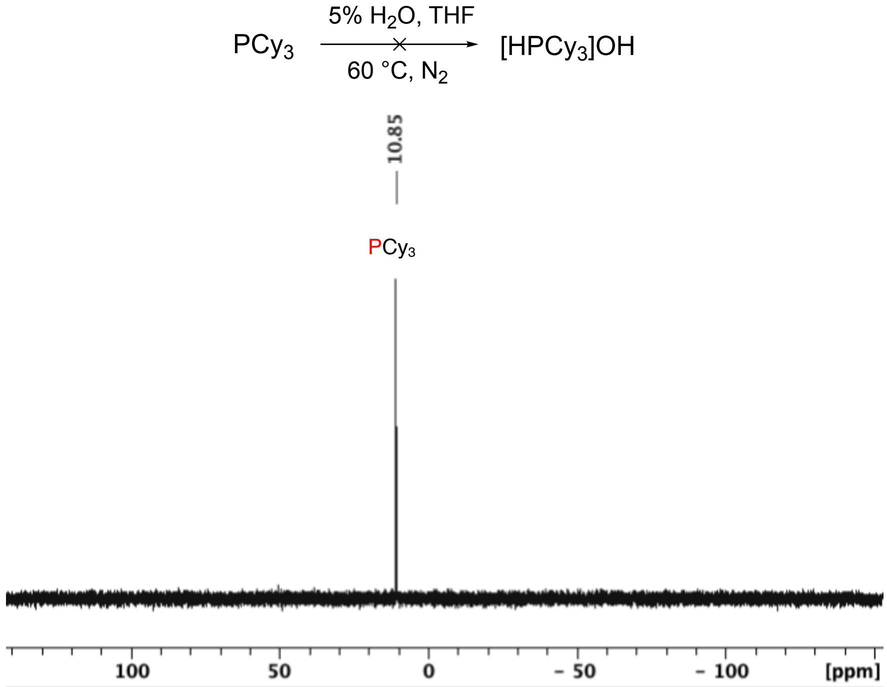

(b) Immediate reaction of PCy_3 with HCl in THF

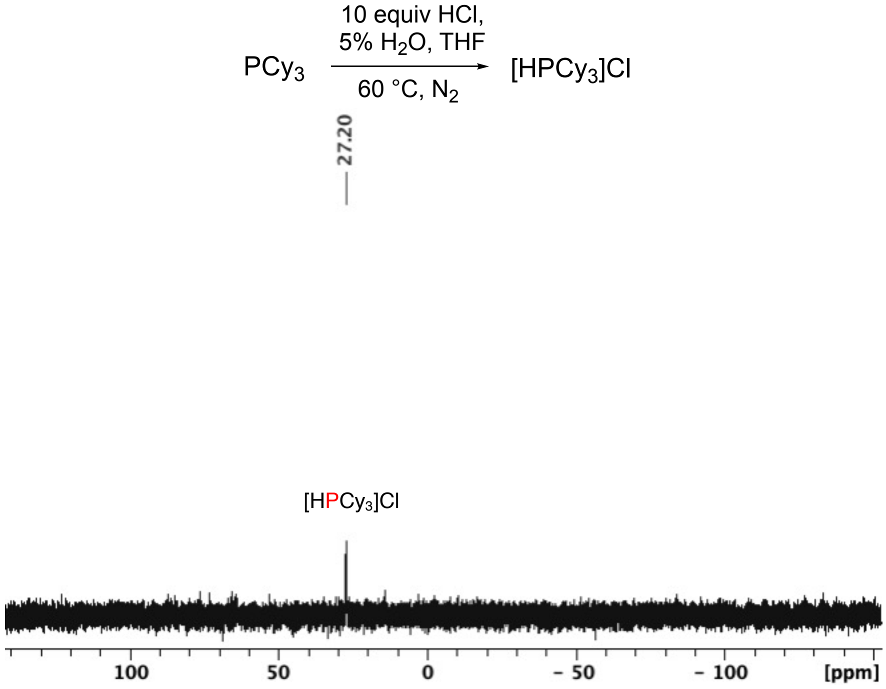

Figure S9. Assessing the reactivity of PCy_3 toward water in THF as solvent by $^{31}P\{^1H\}$ NMR analysis (121 MHz, THF). (a) Spectrum after heating for 24 h at 60 °C in THF containing 5% H_2O (v/v). (b) Immediately after adding concentrated (12 M) HCl.

(a) Stability of PCy_3 to 5% H_2O in C_6H_6

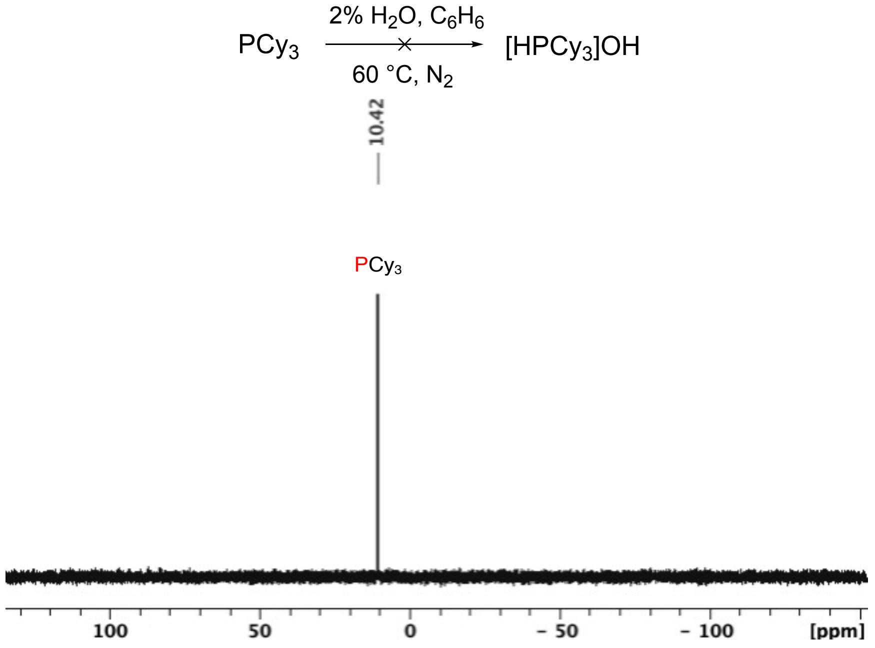

(b) Immediate reaction of PCy_3 with HCl in C_6H_6

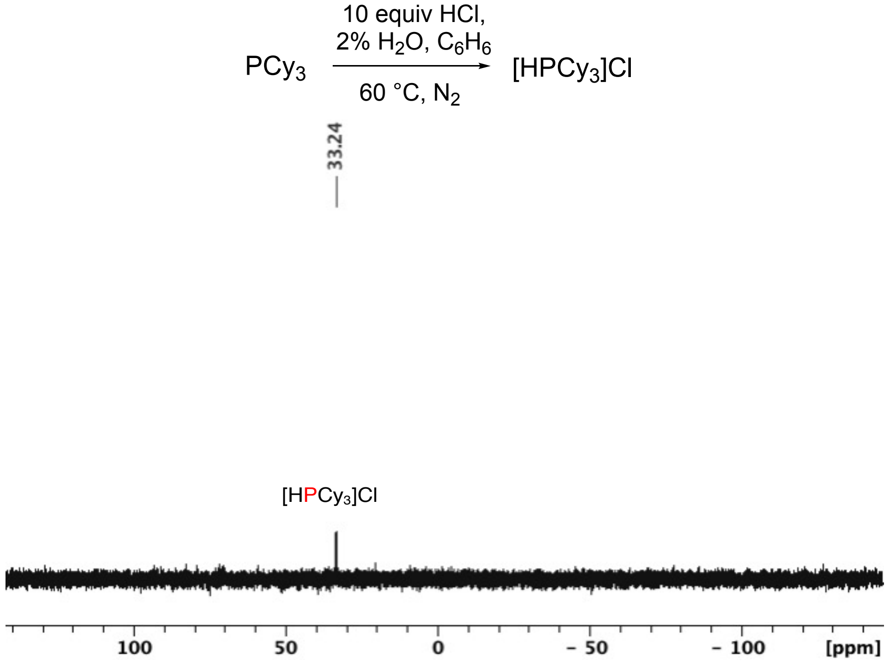

Figure S10. Assessing the reactivity of PCy_3 toward water in benzene as solvent by $^{31}P\{^1H\}$ NMR analysis (121 MHz, THF). (a) Spectrum after heating for 24 h at 60 °C in C_6H_6 containing 5% H_2O (v/v). (b) Immediately after adding concentrated (12 M) HCl.

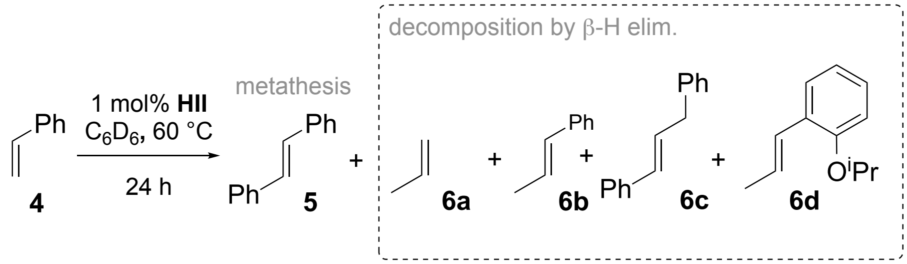

(a) t = 0 h: Prior to adding styrene **4**

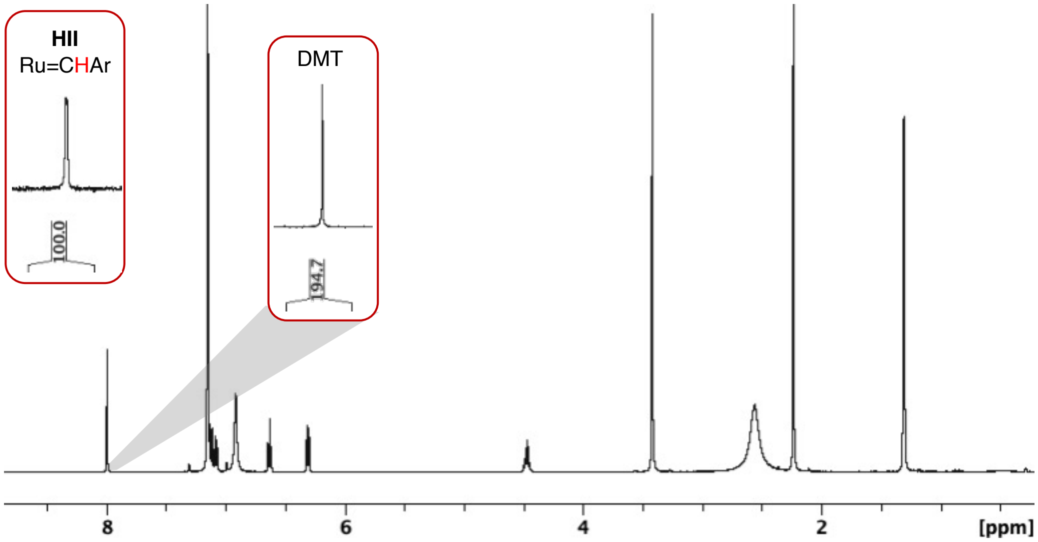

(b) Spectrum 24 h after adding **4**

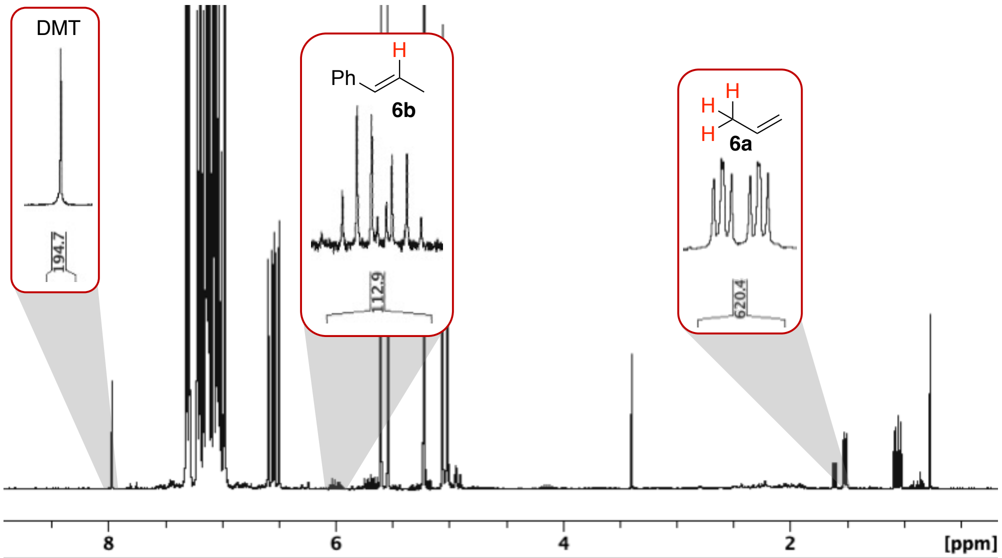

Figure S11. Propene formation in metathesis of styrene by **HII**: quantitation by ^{1}H NMR analysis (500 MHz, C_6D_6). (a) Spectrum prior to adding styrene (the inset shows the integration for the alkylidene proton). (b) Spectrum 24 h after adding styrene. Integrations of key signals (insets) are normalized to that of starting **HII**. Propenes **6c** and **6d** were not observed.

S3. References.

(1) Nascimento, D. L.; Davy, E. C.; Fogg, D. E., Merrifield Resin-Assisted Routes to Second-Generation Catalysts for Olefin Metathesis. *Catal. Sci. Technol.* **2018**, 1535–1544.

(2) Sanford, M. S.; Love, J. A.; Grubbs, R. H., A Versatile Precursor for the Synthesis of New Ruthenium Olefin Metathesis Catalysts. *Organometallics* **2001**, *20*, 5314–5318.

(3) Michrowska, A.; Bujok, R.; Harutyunyan, S.; Sashuk, V.; Dolgonos, G.; Grela, K., Nitro-Substituted Hoveyda-Grubbs Ruthenium Carbenes: Enhancement of Catalyst Activity through Electronic Activation. *J. Am. Chem. Soc.* **2004**, *126*, 9318–9325.

(4) Day, C. S.; Fogg, D. E., High-Yield Synthesis of a Long-Sought, Labile Ru-NHC Complex and Its Application to the Concise Synthesis of Second-Generation Olefin Metathesis Catalysts. *Organometallics* **2018**, *24*, 4551–4555.

(5) Blanco, C.; Nascimento, D. L.; Fogg, D. E., Routes to High-Performing Ruthenium-Iodide Catalysts for Olefin Metathesis: Phosphine Lability Is Key to Efficient Halide Exchange. *Organometallics* **2021**, *40*, 1811–1816.

(6) Varray, S.; Lazaro, R.; Martinez, J.; Lamaty, F., New soluble-polymer bound ruthenium carbene catalysts: Synthesis, characterization, and application to ring-closing metathesis. *Organometallics* **2003**, *22*, 2426–2435.

(7) Blacquiere, J. M.; Jurca, T.; Weiss, J.; Fogg, D. E., Time as a Dimension in High-Throughput Homogeneous Catalysis. *Adv. Synth. Catal.* **2008**, *350*, 2849–2855.

(8) Ferguson, M. L.; O'Leary, D. J.; Grubbs, R. H., Ring-closing metathesis synthesis of N-Boc-3-pyrroline. *Org. Synth.* **2003**, *80*, 85–92.

(9) Bailey, G. A.; Foscato, M.; Higman, C. S.; Day, C. S.; Jensen, V. R.; Fogg, D. E., Bimolecular Coupling as a Vector for Decomposition of Fast-Initiating Olefin Metathesis Catalysts. *J. Am. Chem. Soc.* **2018**, *140*, 6931–6944.
